# Development of message passing-based graph convolutional networks for classifying cancer pathology reports

**DOI:** 10.1186/s12911-024-02662-5

**Published:** 2024-09-17

**Authors:** Hong-Jun Yoon, Hilda B. Klasky, Andrew E. Blanchard, J. Blair Christian, Eric B. Durbin, Xiao-Cheng Wu, Antoinette Stroup, Jennifer Doherty, Linda Coyle, Lynne Penberthy, Georgia D. Tourassi

**Affiliations:** 1 Computational Sciences and Engineering Division, Oak Ridge National Laboratory, 1 Bethel Valley Road, Oak Ridge, Tennessee 37830 USA; 2 College of Medicine, University of Kentucky, Lexington, Kentucky 24105 USA; 3 Louisiana Tumor Registry, Louisiana State University Health Sciences Center, School of Public Health, New Orleans, Louisiana 70112 USA; 4 New Jersey State Cancer Registry, Rutgers Cancer Institute of New Jersey, New Brunswick, New Jersey 08901 USA; 5 Utah Cancer Registry, Huntsman Cancer Institute, University of Utah, Salt Lake City, Utah 84132 USA; 6 Information Management Services, Inc., Calverton, Maryland 20705 USA; 7 Surveillance Research Program, Division of Cancer Control and Population Sciences National Cancer Institute, Bethesda, MD 20814 USA; 8 National Center for Computational Sciences, Oak Ridge National Laboratory, Oak Ridge, Tennessee 37830 USA

**Keywords:** Graph, Graph of words, Graph convolutional networks, Message passing networks, Information extraction, Cancer pathology reports, Deep learning, Natural language processing

## Abstract

**Background:**

Applying graph convolutional networks (GCN) to the classification of free-form natural language texts leveraged by graph-of-words features (TextGCN) was studied and confirmed to be an effective means of describing complex natural language texts. However, the text classification models based on the TextGCN possess weaknesses in terms of memory consumption and model dissemination and distribution. In this paper, we present a fast message passing network (FastMPN), implementing a GCN with message passing architecture that provides versatility and flexibility by allowing trainable node embedding and edge weights, helping the GCN model find the better solution. We applied the FastMPN model to the task of clinical information extraction from cancer pathology reports, extracting the following six properties: main site, subsite, laterality, histology, behavior, and grade.

**Results:**

We evaluated the clinical task performance of the FastMPN models in terms of micro- and macro-averaged F1 scores. A comparison was performed with the multi-task convolutional neural network (MT-CNN) model. Results show that the FastMPN model is equivalent to or better than the MT-CNN.

**Conclusions:**

Our implementation revealed that our FastMPN model, which is based on the PyTorch platform, can train a large corpus (667,290 training samples) with 202,373 unique words in less than 3 minutes per epoch using one NVIDIA V100 hardware accelerator. Our experiments demonstrated that using this implementation, the clinical task performance scores of information extraction related to tumors from cancer pathology reports were highly competitive.

## Introduction

Cancer is a group of chronic diseases. Long-term cancer surveillance, such as monitoring cancer prevalence, treatments, metastasis, and recurrence, is essential to understanding and curing these diseases [1]. In public health contexts, *cancer surveillance* refers to the ongoing collection of information about diagnoses of cancers. Cancers are reportable diseases in the United States [2], and cancer cases are reported to state cancer registries. Monitoring and analyzing cases reported by cancer registries is the most convenient and effective approach to conducting cancer surveillance and research.

Cancer pathology laboratories submit pathology data to cancer registries via electronic cancer pathology reports. The pathology report describes the pathologist’s diagnosis of tissue samples taken from the tumor, including specific information about the characteristics of the tumor [3]. This information includes topography and morphology, a histologic grade that compares the size and shape of cancerous cells to healthy cells, and the stage, which is based on the tumor’s size, location, and spread.

Cancer pathology reports are intrinsically free-form unstructured text documents. Information extraction and annotation from the reports mainly depends on the labor of trained registrars. However, because of the manual nature of this process, information extraction tasks are labor-intensive, costly, and prone to errors. Automatic extraction of information from cancer pathology reports is a cost-effective alternative. Several researchers have studied the possibility of applying machine learning (ML) algorithms to automate information extraction from the pathology reports [3–5].

With the recent advancements of artificial intelligence, deep learning techniques, and natural language processing algorithms, task performance of the information extraction tasks has drastically improved in terms of accuracy [6]. We previously applied both convolutional neural networks (CNNs) [7] and self-attention networks to these tasks [8, 9]. We demonstrated that the deep learning–based models achieve higher task performance scores than traditional ML algorithms.

The volume of cancer pathology reports the model must process is now number in the millions, but the computing capacity of the state cancer registries remains limited. The deep learning models for information extraction should include fast processing and a reasonable level of task performance that is sufficient to replace human annotators. MT-CNNs [10] are by far the most appropriate model for this purpose, but we are pursuing even more capable models in terms of higher accuracy and faster inference time.

The graph-of-words (GOW) representation has been the subject of several research studies [11]. The GOW identifies co-occurrences of words within a specified distance, and then it constructs a graph in which the nodes are words that appear in the document and the edges are co-occurrences of the words. Previously, we applied the bag-of-graphs model to the information extraction tasks [12]. Our study demonstrated that the GOW representation can handle ambiguity of expression variations, such as “word inversion” and “subset matching”. However, with the bag-of-graphs model, the word co-occurrences were treated as a form of bi-grams, which were limited to articulating the higher-order text representations.

Such limitations have been addressed using the graph convolutional network (GCN) model presented in [13]. We applied the GCN to information extraction from the cancer pathology reports [14] and demonstrated that the convolution over GOW nodes is an effective method for information extraction. However, we also identified that the GCN-based text classification [15] possess a limitation when we deploy a trained model.

The newly introduced message passing–based graph convolutional networks (TextLevelGCN) [16] gave us initial encouragement that the message passing network (MPN) can overcome the limitations of the plain GCN. However, we subsequently determined that design of TextlevelGCN is not sufficiently compatible with the hardware accelerator (e.g., graphics processing unit [GPU]). Thus, it is not currently suitable for our information extraction tasks, which focus on applications using millions of training samples.

Our scientific contributions include (1) developing a message passing–based GCN (FastMPN) that is hardware accelerator–friendly; (2) demonstrating that the proposed model can be trained by a large text corpus in a short amount of time compared with the previous architecture [16] that requires several hours; and (3) evaluating the model for information extraction from cancer pathology reports in a high-performance computing environment, which provided better clinical task performance compared to the control [10]. In this paper, we discuss related GCN approaches. In the Methods section, we present the design of the FastMPN, which we evaluate in terms of scalability and clinical task performance in the results. In closing, we provide avenues for future work.

## Related work

A challenge of applying natural language processing models such as GCN is that they can generate extremely large and complex data structures. Analysis of these data structures can generate very large vectors and matrices, which may be sparse or contain a large amount of spurious information [12, 14]. Several approaches have been created during the past 20 years to deal efficiently with graph complexities, and they can be applied to the complexities encountered within GCN models [17, 18]. For example, applications of dimensionality reduction in natural language processing and deep learning are designed to select and orchestrate a set of algorithms that, combined, decrease the dimensions of the original dataset and maintain the basic and core features without oversimplification [19, 20]. Classical approaches to dimensionality reduction include singular value decomposition [21, 22], principal component analysis [23, 24], and CUR decomposition [25, 26]. Other approaches include using the tensor family, such as tensor factorization and the Rank-1 tensor [27]. These dimensionality reduction techniques have been applied to both CNNs [7] and GCNs [13, 15, 28] and have demonstrated improvements in performance. However, the following challenges have been reported for GCNs: (1) GCN models may not completely identify small and simple graphs with homogeneous features and may fail to distinguish proportionally equivalent multi-sets [29], (2) GCN models are susceptible to noise in graph data [30], and (3) major memory bottlenecks may be generated in GCNs [14, 31].

To predict a particular node’s properties or features, GCN approaches examine adjacent nodes and their properties by averaging information from neighboring nodes with information from the individual node. This process is repeated for each node, and this inductive capability allows the same approach to be applied to any new nodes that join the network. Thus the overall graph does not need to be computed again; only the new nodes and subnodes require computing.

One of the first—and perhaps one of the most fascinating—studies to apply a GCN approach is described in [32], where Gilmer et al. present a general framework for supervised learning on graph structured data called *message passing neural networks*, which predicted the quantum mechanical properties of small organic molecules. GCN is currently receiving unprecedented attention because recent studies have demonstrated its superior performance on link prognosis [33] and on several classification tasks focused on both nodes [34] and graphs [35]. In addition, because data are frequently presented in graph-like structures in chemistry, biology, materials science, electronic health records, social networks, and other research areas, GCN is a more natural approach to handling graph-like data.

Development of GCN-based solutions to bio-clinical data has dramatically increased in the past couple of years. Bio-clinical data intrinsically take the form of graph-like structures and can be better represented with graphs. Several examples of studies of GCN can be applied to bio-clinical data: an approach that uses GCN for images of chest x-rays for disease identification and localization [36]; a deep voxel-graph convolution network model tested on 3D positron-emission tomography images to predict a patient’s lung cancer stage, compared with classical 3D CNN (with image padding) and radiomics models [37]; the application of graph CNN to gene expression data for breast cancer patients to predict the occurrence of metastatic events[38]; and the development of a GCN method for discovering non–small cell lung cancer complexity in immuno-oncology treatment responses based on high-dimensional electronic health records and genomic data [39], among others.

Approaches based on GCN have developed rapidly in the past few years. One factor that appears constant is the large number of models developed on GCNs that claim to be superior to other models. However, we found it difficult to assess the new models because there are no official benchmarks for analyzing GCN models. This is not an isolated observation, and because of this opportunity, researchers have developed comparative approaches. A couple of examples of these are reference [40], which performed a classification of the approaches, and reference [41], which presented a comparative study for systematic comparison.

## Methods

### Cancer pathology report datasets

The dataset for this study consisted of unstructured text in pathology reports from four cancer registries: the Kentucky Cancer Registry, Louisiana Tumor Registry, New Jersey State Cancer Registry, and Utah Cancer Registry. These registries participate in the National Cancer Institute’s (NCI) Surveillance, Epidemiology, and End Results (SEER) program. The study was executed in accordance with the institutional review board protocol DOE000152.

We determined truth labels of the pathology reports based on the Cancer/Tumor/Case, which stores all diagnostic, staging, and treatment data for a reportable neoplasm in the NCI SEER Data Management System. We considered the International Classification of Diseases for Oncology, Third Edition, coding convention in labeling the cases. We extracted information for six clinical tasks from each cancer pathology report, including the number of class labels per task: main site (70 classes), subsite (324 classes), laterality (7 classes), histology (572 classes), behavior (4 classes), and histological grade (9 classes).

Note, the cancer pathology reports are essentially free-form natural language text, which allows linguistic variability of expression. For example, there are several ways to express histologic grade 3 (poorly differentiated). The following list is a small sample of the variations we identified in the corpus:

- “histologic grade: 3”
- “histologic grade (mbr) 3”
- “histologic grade (g1-3): 3”
- “histologic grade: g3”
- “histologic grade: poorly differentiated”
- “histologic grade: differentiated poorly”
- “histologic grade: grade iii”
- “histologic grade: high grade (poorly differentiated)”

### Graph of words representation

The most intuitive way to identify the variability of expressions is to learn them as they are stated. This approach is practical if we provide rich datasets that include all possible variations. Using this approach, the model may not make the correct judgment if it encounters a new mutation of expressions. In the study, we supplied 667,290 cases to train the model, so we may be able to field most of the ambiguity and variability within. However, we are applying more effective means of describing linguistic variabilities in the free-form text corpus.

We adopted GOW as a representation of the natural language text in the reports. The graph nodes are unique words in the documents, and edges represent co-occurrences between the words within distance *d*. The GOW representation provides flexibility and robustness in describing natural language texts. Suppose we describe the expressions of the histologic grade 3 listed earlier. We can reduce the variability due to the word ordering (e.g., poorly differentiated and differentiated poorly) and additive terms (e.g., grade 3 and grade [g1-3] 3).

**Fig. 1:**
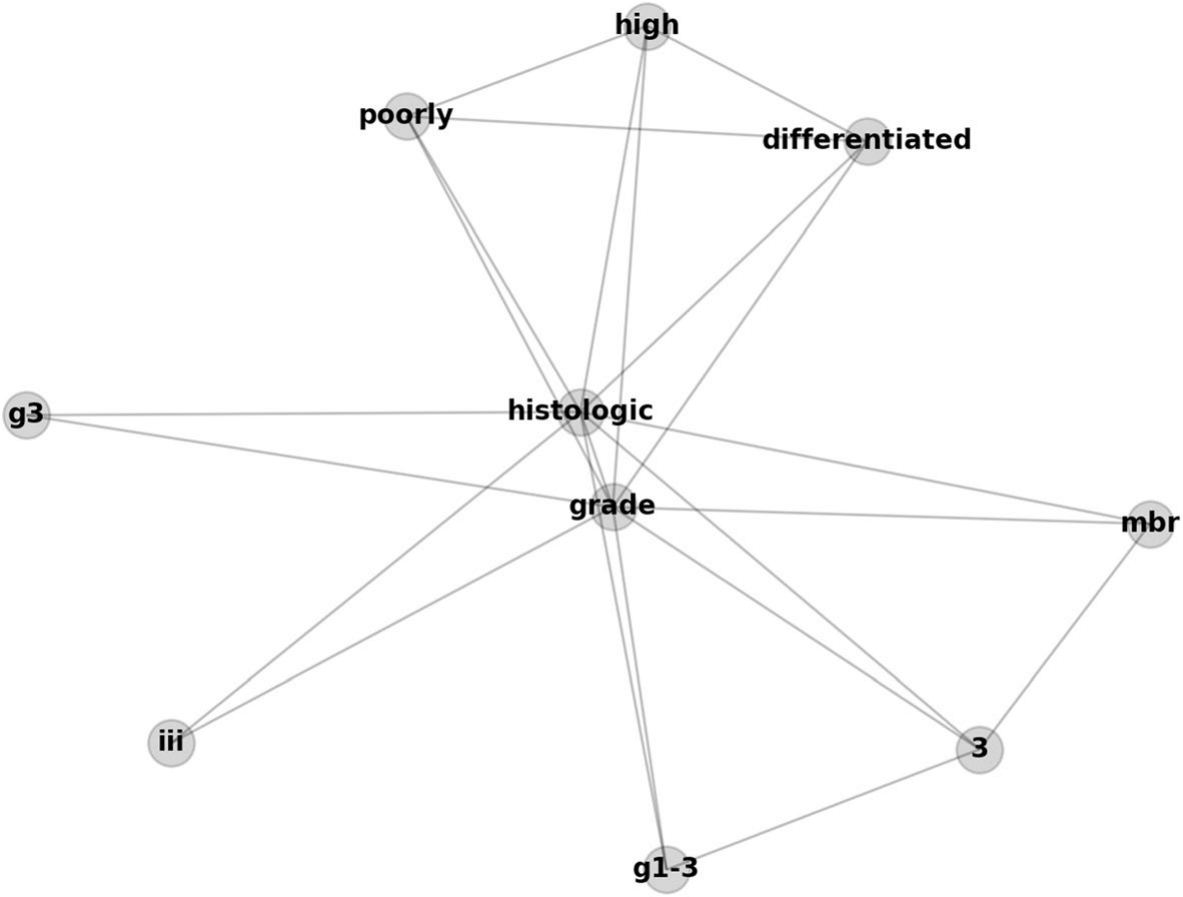
A sample GOW. The GOW by the list of sample phrases describing the histologic grade 3 (poorly differentiated)

Applying graph convolution layers on GCN will provide another level of abstraction. The TextGCN implementation generates a graph with words as the nodes, and the edges between the nodes are co-occurrences of the words at the corpus level. Figure 1 is the illustration of the GOW of the histologic grade 3 listed above. Training TextGCN is performed by finding the optimal graph embeddings of the nodes, which maximizes the document classification accuracy.

### Graph convolutional networks for text comprehension

The studies described herein focus on the application of GCN-based text classification models. The selection of GCN models was strongly influenced by the capabilities of the models to accept graphs as a feature representation in an “as found” state and to use those graphs to perform classification tasks leveraging GCN capabilities. GCN is a member of the CNN family and performs similar operations in that it learns features from neighbors. The major difference between the two is that a CNN is designed to operate on spatial data, whereas a GCN works with nodes in cases the adjacent nodes are not necessarily close in Euclidean distance.

Yao et al. [15] designed TextGCN, a variant of GCN, to solve document classification problems. Essentially, they imported documents and associated words in the corpus as nodes in the graph and established connections between nodes. The forward pass equation of the GCN is

$$\begin{aligned} \textbf{H}^{l+1}=\sigma ( \textbf{A} \cdot \textbf{H}^{l} \cdot \textbf{W}^{l} ), \end{aligned}$$

where $\textbf{H}^{l}$ is the feature representation of the nodes at layer *l*, $\textbf{A}$ is the adjacency matrix, and $\textbf{W}^{l}$ is the node embedding matrix at layer *l*. Note that if $l=0$, then $\textbf{H}^0$ equals the input feature $\textbf{X}$, $\textbf{H}^0=\textbf{X}$. Yao defined edge weights in $\textbf{A}$ with a term frequency-inverse document frequency (TF-IDF) between a document node and a word node and point-wise mutual information (PMI) between word nodes. One interesting factor in Kipf’s GCN and Yao’s TextGCN design is that the edge weights in the adjacent matrix ${\textbf {A}}$ remain constant throughout the training and inference; that is, the node embedding matrix $\textbf{W}$ is the only trainable parameter.

The inference of a GCN is the summation of influences of adjacent nodes of precedent layers. Note that Yao’s TextGCN approach adopts the GCN mechanism by performing the training and testing of text documents and words in one graph. It implies that the data samples must be in the graph if a classifier is implemented based on GCN. However, two problems may occur if we employ Yao’s TextGCN for the task of cancer surveillance.

First, the graph must include words and documents altogether. Suppose we are developing a TextGCN model with 1 million cancer pathology reports, and the corpus has 100,000 words in the vocabulary. The size of the adjacency matrix $\textbf{A}$ becomes 1,100,000 $\times$ 1,100,000. Based upon the definition of $\textbf{A}$ in [15], the document-document portion of $\textbf{A}$ is simply a 1,000,000 $\times$ 1,000,000 identity matrix, which is highly inefficient.

Second, the GCN only determines the inference of data samples already in the graph, but we cannot make inferences from the newly introduced documents. Thus, developing a TextGCN model with data from one cancer registry and deploying the model to other registries whose data has not been included in the model, or even not allowed to be exposed, would result in an infeasible TextGCN model.

### Message passing architecture

The TextLevelGCN developed by Huang et al. [16] suggested a new GCN model that allowed edge weights to be trainable as well. The model employed a message passing architecture that collected information from adjacent nodes and updated the node embeddings as follows:

1 $$\begin{aligned} \textbf{M}_n = \underset{a \in N_n^p}{\max }\ e_{an} \textbf{r}_a, \end{aligned}$$

where $\textbf{M}_n$ represents the messages the node *n* receives from its neighbors $\textbf{r}_a$, $e_{an}$ is the edge weight from node *a* to node *n*, and $N_n^p$ denotes the nodes that represent the nearest *p* words of *n* in the text. Note, $\max$ combines the maximum values in each dimension. The node embedding is updated using

$$\begin{aligned} \textbf{r}_n^{new} = (1- \eta _n) \textbf{M}_n + \eta _n \textbf{r}_n^{old}, \end{aligned}$$

where $\eta _n$ is a trainable variable for node *n* that indicates how much information from $r_n^{old}$ should be kept and transferred to $r_n^{new}$. The class label of the text is predicted using

$$\begin{aligned} y = softmax \left( \sigma \left( \textbf{V} \sum r_n + b \right) \right) , \end{aligned}$$

which is a summation of the node embeddings of the words appearing in the document, where $\textbf{V}$ is a matrix that maps the vector space into the output space, *b* is bias, and $\sigma (\cdot )$ is an activation function.

One substantial departure from the TextGCN is that the TextLevelGCN does not convey document nodes in the model, which is a highly desirable feature if we want to deploy the trained model to our partners and the public.

### Fast message passing networks

Optimally, the computation should maximize the utility of the parallel computational units available for matrix multiplication, but two things we identified from the implementation of MPNs in the TextLevelGCN model make that difficult. First, in the Eq. (1), $\max$ operation is not well suited for parallel computation and updating. In the implementation of [16], the computation was done on a node-by-node basis, which results in a slow computation even with the GPU.

Second, in the MPN, both the embedding matrix and weight matrix become trainable. The adjacency matrices of word co-occurrences are typically sparse. Hence, the best practice is to implement the MPN using sparse matrix representations and computations to save the memory of computational hardware accelerators. Implementations of sparse matrices are supported by scientific computing libraries, artificial intelligence, and deep learning environments. However, the sparse matrix representation on the PyTorch platform as a form of a trainable variable has not been implemented to date.

Our implementation of FastMPN applied a modification to the previous architecture [16], and a hardware accelerator–friendly approach. Rather than taking the maximum value of the neighborhood and then updating the node embedding, we applied a weighted sum over a given node and neighborhood to generate the update:

2 $$\begin{aligned} \textbf{M}_n^{\prime } = \sum \limits _{i=0}^{N} e_{in} \textbf{r}_i, \end{aligned}$$

where *N* is the number of nodes(equal to the number of words from the corpus) in the model, and means the message to node *n*, $\textbf{M}_n^{\prime }$ is the summation of the neighbors $\textbf{r}_i$ with the weight from node *i* to node *n*, $e_{an}$. The globally shared edge weights determine the importance of a given token in relationship to its neighbors through the sum.

We further optimized the FastMPN based on the PyTorch geometric platform by indexing the matrix elements to collect the necessary variables for the forward- and backward-pass, arranging a matrix into a 1D array to apply hardware-accelerated computations:

3 $$\begin{aligned} \textbf{M}_n^{opt} = \sum \limits _{a \in N_n^p} e_{an} \textbf{r}_a, \end{aligned}$$

where $\textbf{M}_n^{opt}$ is the messages the node *n* receives from its neighbors $\textbf{r}_a$, $e_{an}$ is the edge weight from node *a* to node *n*. Note, because the calculation of $\textbf{M}_n^{opt}$ uses a collection of necessary variables, $\textbf{M}_n^{opt}$ should be equivalent to $\textbf{M}_n^{\prime }$, $\textbf{M}_n^{opt}=\textbf{M}_n^{\prime }$, but requires less computational resources to calculate.

## Results

The FastMPN model code was developed using PyTorch 1.4 [42] and Python 3.6 [43], which are available on the IBM Watson Machine Learning platform. We used the PyTorch Geometric library [44] for implementing the graph convolutional networks and Horovod [45] for data parallelism. The scalability and clinical task performance evaluations were performed on the Summit supercomputer, which is operated by the Oak Ridge Leadership Computing Facility.

### Scalability in data parallelism

In the case of the cancer pathology reports classification, suppose we applied window size $d=2$ and word embedding vector length $w=300$; the FastMPN model requires 69,629,027 training parameters, where 60,711,900 parameters belong to the word embedding, which is nearly 90% of the total parameters. One aspect of the embedding layers is not favorable for data parallelism: the embedding layers require a substantial memory block but do not consume much computational power. In terms of data parallelism, this characteristic is not beneficial because the embedding layers increase the communication demand, reduce the time for computation, thus decreasing the throughput.

We performed experiments to measure the training time (seconds) per epoch with data parallelism of the FastMPN models. For a given mini-batch size, either 512 or 10,240, we applied data parallelism with 1, 4, 8, 16, and 32 GPUs. The elapsed training time per epoch of 667,290 training samples for each data parallelism are listed in Table 1. Note that the result with the mini-batch size 10,240 and one GPU is not available because it was unable to execute because of the limited capacity of GPU memory.

**Table 1:** Elapsed training time (seconds) per epoch of the FastMPN model with 667,290 training samples of cancer pathology reports, with the mini-batch size set to either 512 or 10,240. Experiments were conducted with trainable or fixed-embedding layers. Data parallelism was implemented with the Horovod library and executed on the Summit supercomputer

Mini-batch size	512		10,240	
GPU’s	trainable	fixed	trainable	fixed
1	154.78	138.91	N/A	N/A
4	87.73	58.84	57.26	44.57
8	73.72	41.52	31.49	23.30
16	74.65	35.70	20.43	13.22
32	68.89	28.65	13.34	9.07

We observed that the elapsed time decreased as more GPUs were supplied. The decrease was more significant for the fixed embedding models, which is expected because the communication overhead was reduced to the fixed embedding ones. Likewise, experiments with the mini-batch size 10,240 recorded faster than with the mini-batch size 512. Smaller mini-batch sizes resulted in more frequent backward passes, requiring more communication between nodes and GPUs.

We decided to use one GPU for the following clinical task performance experiments because the required training time per epoch was less than 3 minutes, which is an affordable amount of time to spend.

### Clinical task performance

We evaluated the clinical task performance of extracting the six task labels (site, subsite, laterality, histology, behavior, and grade) of the primary tumor described in the cancer pathology reports. We employed both micro- and macro-averaged F1 scores, because the tasks possess severe class imbalance. The micro-averaged F1 scores are useful if we observe the task performance for each document, whereas the macro-averaged F1 scores may reflect better toward the minority class labels.

We are aware that it could be confusing to compare model performances with 12 scores (6 tasks, macro- and micro-F1 scores per each task) together. Therefore, we applied a numerical average of those 12 scores then reported to the rightmost column in Table 2. Note, the average numbers are for comparison purposes only and may not convey any clinical implications.

**Table 2:** Clinical task performance of the FastMPN models with various choices of hyper-parameters (dropout, word distance, and mini-batch size) in micro- and macro-averaged F1 scores

**Arch**	**Drop**	**d**	**Batch**	**Site**		**Subsite**		**Laterality**		
				**Micro**	**Macro**	**Micro**	**Macro**	**Micro**	**Macro**	
MT	0.0	2	64	0.9292	0.6553	0.6552	0.2869	0.9149	0.5162	
MT	0.0	2	256	0.9260	0.6727	0.6444	0.3008	0.9096	0.5149	
MT	0.0	5	64	0.9298	0.6762	0.6521	0.3072	0.9145	0.5159	
MT	0.0	5	256	0.9267	0.6747	0.6424	0.3069	0.9098	0.5141	
MT	0.0	10	64	0.9277	0.6704	0.6528	0.2981	0.9156	0.5173	
MT	0.0	10	256	0.9279	0.6696	0.6464	0.3097	0.9113	0.5254	
MT	0.25	2	256	0.9306	0.6738	0.6548	0.3073	0.9147	0.5136	
MT	0.25	5	256	0.9307	0.6834	0.6534	0.2967	0.9145	0.5081	
MT	0.25	10	256	0.9306	0.6768	0.6548	0.3049	0.9150	0.5204	
ST	0.25	10	256	0.9310	0.6571	0.6532	0.3045	0.9143	0.5085	
**MT-CNN**										
MT	0.25	N/A	256	0.9250	0.6754	0.6538	0.3208	0.9000	0.5083	
**Arch**	**Drop**	**d**	**Batch**	**Histology**		**Behavior**		**Grade**		**Average**
				**Micro**	**Macro**	**Micro**	**Macro**	**Micro**	**Macro**	
MT	0.0	2	64	0.7781	0.2913	0.9730	0.8873	0.7642	0.6197	0.6893
MT	0.0	2	256	0.7752	0.3219	0.9726	0.8907	0.7467	0.6411	0.6931
MT	0.0	5	64	0.7782	0.3197	0.9731	0.8899	0.7624	0.6538	0.6977
MT	0.0	5	256	0.7783	0.3240	0.9735	0.8909	0.7536	0.6556	0.6959
MT	0.0	10	64	0.7788	0.3137	0.9728	0.8778	0.7654	0.6677	0.6965
MT	0.0	10	256	0.7765	0.3363	0.9720	0.8547	0.7541	0.6380	0.6935
MT	0.25	2	256	0.7798	0.2949	0.9737	0.8758	0.7649	0.6726	0.6964
MT	0.25	5	256	0.7792	0.3073	0.9739	0.8932	0.7630	0.6608	0.6970
MT	0.25	10	256	0.7802	0.3391	0.9739	0.8925	0.7627	0.6653	0.7014
ST	0.25	10	256	0.7785	0.3183	0.9739	0.8797	0.7567	0.6600	0.6946
**MT-CNN**										
MT	0.25	N/A	256	0.7651	0.3642	0.9652	0.8700	0.7401	0.6345	0.6935

We reported the following six information extraction tasks from the cancer pathology reports: cancer main site, subsite, laterality, hisology, behavior, and grade. MT stands for multi-task learning, ST means the single-task learning mechanism. Owing to the difficulty of comparing performance of models with respect to the 6 tasks and 12 scores, we added 1 column on the right filled with the arithmetic average of all 12 F1 scores. The score may not have a relevant clinical implication, but it helps intuitively compare model performances

#### Word distance

Word distance *d* plays an essential role in describing the variability of expressions to the GOW representations, such as “histologic grade: poorly differentiated” and “histologic grade: high grade (poorly differentiated).” However, setting *d* high widens the window for determining the co-occurrence of words and slows the graph-building process. Moreover, it may introduce unnecessary edges in the GOW and may degrade classification performance.

The results in Table 2 reported that if we applied a dropout of 0.25, a wider co-occurrence window resulted in a higher score (0.7014) than (0.6964), implying that more connection between words of longer distance provided a better chance of capturing useful features to make sound judgments. On the other hand, if we applied a dropout of 0.0 (no dropout), we observed the longer word distance $d=10$ scored lower (0.6935) than $d=5$ (0.6959), which means the wider co-occurrence window also introduced noisy connection of words. It also implies the dropout regularization was useful to avoid overfitting and improved the clinical task performance.

#### Single-task vs. multi-task learning mechanism

A multi-task (MT) learning mechanism was designed to let one model solve multiple tasks with related, but not orthogonal, tasks. The purpose of MT learning is that, by solving several associated problems altogether, the model could possess generalizable feature sets rather than the single-task (ST) mechanism, thus improving overall classification accuracy conjointly. We implemented the MT by appending additional fully connected layers per task. It may increase the number of trainable parameters slightly, but it learns and solves six tasks simultaneously, saving computational resources and time overall.

We compared the ST and MT versions of the FastMPN with $d=10$, a mini-batch size of 256, and a dropout of 0.25. The MT scored 0.7014, and the ST achieved 0.6946. These results suggest that MT learning is a good investment in both computational efficiency and task performance.

## Discussion

In our implementation of the FastMPN model, we introduced a simplified yet hardware accelerator–friendly design. This model achieved training with several hundred thousand text samples in a reasonable amount of time compared to the message passing TextLevelGCN implementation by Huang et al. [46], which involved a training time that was too long to be practical.

Scalability in data parallelism is not trivial for natural language text models because a large number of trainable parameters should be carried with the word embedding layer. The embedding layer requires less computation but more communication, and thus degrades the throughput of the data parallelism. This phenomenon is similar to the node embedding layer in the FastMPN model. As shown in Table 1, with the single GPU, the fixed-embedding model requires slightly less training time per epoch than the one with trainable embedding (154.78 vs. 138.91). On the contrary, for the data parallel training with 32 GPUs, the fixed-embedding model performed more than two times faster (68.89 vs. 28.65).

The choice of word distance *d* affected the clinical task performances. Longer distance increased the acceptance of linguistic variability to the GOW, thus improving the classification accuracy scores. However, a distance that is too long also increased the chance of adding unnecessary node connections, resulting in decreased performance. Adding dropout regularization helped to mitigate the overfitting and improved task performance.

Overall, the FastMPN performed as well as or better than the MT-CNN model [10] (0.7014 vs. 0.6935). The improvement was notable for the tasks involving site, laterality, behavior, and grade. We also observed notable improvements for tasks with fewer class labels. On the other hand, with the tasks of subsite and histology, which have more than 300 class labels, the MT-CNN scored higher macro-F1 scores than the FastMPN. The latter result indicates that, even though the FastMPN still made the correct case-level decisions compared to the MT-CNN for classifying subsite and histology labels, the FastMPN did not work very well with the minor class labels in severely imbalanced tasks with many underrepresented classes. This finding suggests a need for further investigation and improvements to the FastMPN models and training protocol.

## Conclusion

This paper presented a hardware accelerator–friendly MPN model for natural language texts based on the message passing architecture, followed by an evaluation of the model through application to the tasks of clinical information extraction from free-form cancer pathology reports. Implementation of the FastMPN model handled nearly 700,000 training samples with more than 200,000 vocabularies using 1 GPU. We have tested performance of the proposed FastMPN model against TextLevelGCN and MT-CNN models using IBM Power System AC922 compute nodes with NVIDIA V100 GPU installed on the Summit supercomputer. The FastMPN model achieved a training time of less than 3 minutes per epoch, whereas the TextLevelGCN required several hours per epoch to train. Finally, we confirmed that the clinical task performance of the FastMPN model was equivalent to or better than that of the MT-CNN model. Further comparisons against against other feasible models are potential topics for future study.

## Data Availability

The data underlying this article was provided by the state cancer registries by permission and cannot be shared publicly due to the privacy of individuals in the data corpus.

## Abbreviations

CNN: Convolutional neural network
GCN: Graph convolution network
GOW: Graph-of-words
GPU: Graphics processing unit
ML: Machine learning
MPN: Message passing network
MT: Multi-task
MT-CNN: Multi-task convolutional neural network
NCI: National cancer institute
PMI: Point-wise mutual information
SEER: Surveillance, epidemiology, and end results
ST: Single-task
TF-IDF: Term frequency-inverse document frequency
